# Why Dieters Succeed or Fail: The Relationship Between Reward and Punishment Sensitivity and Restrained Eating and Dieting Success

**DOI:** 10.3389/fpsyg.2021.636432

**Published:** 2021-05-13

**Authors:** Nienke C. Jonker, Elise C. Bennik, Peter J. de Jong

**Affiliations:** Department of Clinical Psychology and Experimental Psychopathology, University of Groningen, Groningen, Netherlands

**Keywords:** executive control, dieting success, punishment sensitivity, reward sensitivity, restrained eating

## Abstract

**Background:**

The current study set out to improve our understanding of the characteristics of individuals who are motivated to restrict their food intake yet who nevertheless fail to do so. We examined whether punishment sensitivity (PS) was related to restrained eating, and reward sensitivity (RS) to perceived dieting success. Additionally, it was examined whether executive control (EC) moderates the association between RS and perceived dieting success.

**Methods:**

Female student participants (*N* = 290, aged 17–29, BMI between 18.5 and 38.0) completed questionnaires on restrained eating, perceived dieting success, RS and PS, and carried out a behavioral task to index EC.

**Results:**

PS was indeed positively related to restrained eating. RS was positively related to perceived dieting success, yet, EC did not moderate this association.

**Conclusion:**

The current study adds to the evidence that PS is related to individuals’ motivation to restrict their food intake. Furthermore, it shows support for the suggestion that RS may *facilitate* food restriction.

## Introduction

Many people attempt to decrease their weight by restricting their food intake (Wardle et al., 2006; De Ridder et al., 2014). However, they often fail to adhere to their diet (Knäuper et al., 2005), or they regain their weight after initially having lost it (Field et al., 2007). With the worldwide prevalence of obesity increasing to nearly triple of the prevalence in 1975 (World Health Organisation, 2018), it seems to be paramount to improve our understanding of the characteristics of individuals who are motivated to restrict their food intake to lose weight (i.e., restrained eaters), and more specifically of those individuals who nevertheless fail to do so. The current study focuses on two candidate traits, reward and punishment sensitivity (Gray, 1970; Gray and McNaughton, 2000), that have been proposed to play a role in eating behavior (e.g., Verbeken et al., 2012; De Decker et al., 2016).

Individuals who are sensitive to punishment are thought to show more avoidance behavior in response to cues of punishment, respond more negatively to punishment, and have more attention to cues of punishment (Gray, 1970; Gray and McNaughton, 2000). High punishment sensitive (PS) individuals might be more sensitive to the punishing consequences of overeating such as becoming fat, and might therefore be more likely to engage in attempts to restrict their food intake to prevent undesirable outcomes (Jappe et al., 2011). Indeed higher PS has been found to relate to a stronger fear of becoming fat and more concern for dieting (Dalley, 2016), and PS was found to be higher in individuals with anorexia nervosa who are characterized by extreme food restriction (Harrison et al., 2010; Jonker et al., 2020). Furthermore, positive associations have been found between PS and restrained eating as measured with the Dutch Eating Behavior Questionnaire (DEBQ; van Strien et al., 1986) and as measured with the Restraint Scale (Herman and Polivy, 1975) in both adolescents and young adults (Ahern et al., 2010; Matton et al., 2013; Jonker et al., 2016a; Matton et al., 2017b). The restrained scale and the DEBQ both seem to identify individuals with an intention to restrict their food intake and seem to have good internal consistency (Herman and Polivy, 1975; van Strien et al., 1986; Johnson et al., 2012). Thus, PS seems to be related to individuals’ motivation to restrict their food intake.

The relationship between reward sensitivity (RS) and food restriction seems less straightforward, and seemingly opposing theories have been proposed about the role in food restriction (e.g., Sala et al., 2018). Individuals who are sensitive to reward are thought to show more approach behavior in response to cues of reward, respond more positively to reward, and have more attention to cues of reward (Gray, 1970; Gray and McNaughton, 2000). On the one hand, RS has been implicated in unsuccessful food restriction (Davis and Woodside, 2002). It is suggested that heightened RS increases the rewarding properties of food thereby *impeding* food restriction. Another possibility is that, as a consequence of overeating the rewarding feeling obtained from food decreases and the amount of food needed to obtain the same rewarding effect increases, thereby *impeding* food restriction (cf., Davis and Fox, 2008; Oginsky and Ferrario, 2019). On the other hand, RS has been implicated in successful food restriction (Bergh and Södersten, 1996). It is suggested that heightened RS increases the rewarding value of the effects of food restriction such as weight loss and improved body shape thereby *facilitating* food restriction. Thus, a positive as well as a negative relationship between RS and food restriction have been suggested. In line with this, studies have failed to find consistent evidence for differential RS in adolescents and young adults with anorexia nervosa (Harrison et al., 2010; Matton et al., 2017a; Jonker et al., 2020), and in adolescents and adults with obesity (Davis and Fox, 2008; Jonker et al., 2016b). Furthermore studies on the relationship between RS and restrained eating show a mixed pattern (Ahern et al., 2010; Stapleton and Whitehead, 2014; Jonker et al., 2016a; Walther and Hilbert, 2016; Matton et al., 2017b). For example, a previous study using the restraint scale to measure restrained eating failed to find an association with RS in young adults (Jonker et al., 2016b). Yet, another study using the DEBQ to measure restrained eating did find a positive association between RS and restrained eating in young adults (Ahern et al., 2010), although a later study failed to replicate this finding in adolescents (Matton et al., 2017b).

Two considerations might help in clarifying the role of RS in food restriction. A first potentially important consideration that has been overlooked is that RS might not so much be involved in individuals’ motivation to restrict their food intake in general, but may specifically contribute to individuals’ success in their attempts to restrict their food intake. This consideration follows from the theories on the role of RS in food restriction suggesting either a facilitating or impeding role and not a general motivating role (Bergh and Södersten, 1996; Davis and Woodside, 2002). The restrained scale and the DEBQ seem to identify individuals with an intention to restrict their food intake, however, both scales might not adequately differentiate between successful and unsuccessful dieters (Johnson et al., 2012). As a consequence, the relationship between RS and individuals’ dieting success cannot be examined by looking at the association between RS and restrained eating as measured with these scales. Therefore, the aim of the current study was to examine the relation between RS and experienced dieting *success* of individuals who have an intention to diet. Based on the proposed theories both a positive as well as a negative association might be expected between RS and dieting success.

A second potentially important consideration is that executive control (EC) might play a role in the relationship between RS and successful food restriction (cf. Jonker et al., 2014). According to dual-process models, behavior results from the interplay of bottom-up processes such as RS related automatic approach responses and top-down control processes (i.e., EC) that guide behavior towards intentions and goals (Strack and Deutsch, 2004). Individuals with strong top-down control, thus individuals who are high in EC, are expected to be able to resist short-term rewards to support the pursuit of long-term goals (Baumeister et al., 2007; Hofmann et al., 2012). Consequently, the proposed *impeding* role of RS in dieting success, implicated in increasing the short-term rewarding value of food, might only be found in individuals with low EC. Individuals with high EC might be able to resist the short-term reward value of food in an effort to pursuit their long-term dieting goal. This moderating role of EC would not be expected in the proposed *facilitating* role of RS in dieting success since in this case the RS related behavior is in line with the long-term goals. Previous studies, albeit with conflicting findings, have examined the extent to which EC moderates the relationship between RS and restrained eating (Jonker et al., 2016a; Matton et al., 2017b), yet this relationship has not been examined with regard to dieting success specifically. Therefore, the current study will examine the extent to which the association between RS and dieting success is moderated by EC.

In short, the main aim of the current study is to examine the relationship between PS and restrained eating, and between RS and restrained eaters’ dieting success. Since there are seemingly opposing theories about the relation between RS and dieting success (Bergh and Södersten, 1996; Davis and Woodside, 2002; Sala et al., 2018), and empirical findings thus far do not seem to show evidence in favor of either, we will explore both as equally likely possibilities. We will examine whether (1) PS is related to individuals’ motivation to restrict their food intake, and expect a positive association between PS and restrained eating behavior, and (2) RS is related to dieting success of individuals with an intention to diet. We explore whether (2a) RS *facilitates* food restriction, implying a positive association between RS and restrained eaters’ dieting success, and/or (2b) RS *impedes* food restriction, implying a negative association between RS and restrained eaters’ dieting success. The negative association between RS and dieting success (2b) is only expected to be found in individuals low in EC.

## Method

### Participants

Initially, 341 female undergraduate students completed this study. Since dieting behavior is more common in women (Wardle et al., 2006; De Ridder et al., 2014), and the relationship between dieting behavior and reward and punishment sensitivity seems to differ between men and women (e.g., Walther and Hilbert, 2016), data of male participants was not analyzed (*n* = 120, see section “Procedure”). Since we are interested in unsuccessful dieting related to the development and maintenance of overweight and obesity, females with underweight [Body Mass Index (BMI) < 18.5] were also excluded (*n* = 26).

### Materials

#### Reward and Punishment Sensitivity

Reward and punishment sensitivity were indexed by the Behavioral Inhibition Scale/Behavioral Activation Scale (BIS/BAS; Carver and White, 1994). The BIS/BAS contains 24 items answered on a 4-point scale ranging from “*Very false for me*” (1) to “*Very true for me*” (4). In line with our previous study (Jonker et al., 2016a), the punishment sensitivity subscale (PS, e.g., “I worry about making mistakes”), the reward responsivity subscale (RR, e.g., “When good things happen to me, it affects me strongly”), and the reward drive subscale (RDr, e.g., “I go out of my way to get things I want”) were reported. Subscale scores are the average scores of the relevant items. Internal consistency of the punishment sensitivity and reward drive subscales were acceptable to good (Cronbach’s alpha of 0.78 and 0.81, respectively). Internal consistency of the reward responsivity subscale was questionable (Cronbach’s alpha of 0.61). These internal consistency scores are very similar to those reported in previous studies (Matton et al., 2013; Jonker et al., 2016a). Since leaving an item out did not improve internal consistency of the RR, as well as for reasons of consistency within the field the average score was used.

#### Restrained Eating

The Restraint Scale (Herman and Polivy, 1975) was used to measure restrained eating. The questionnaire contains ten items answered on a 4-point or 5-point scale (e.g., “How often are you dieting?”). Total scores can range from 0 to 35 and internal consistency in the current study was acceptable (Cronbach’s alpha of 0.77), and comparable to previous studies (Neimeijer et al., 2013; Jonker et al., 2016a).

#### Dieting Success

Given the cross-sectional nature of the current study and the interest in general dieting tendencies (vs. a specific dieting attempt) we assessed subjective dieting success with the Perceived Self-Regulatory Success in Dieting Scale (PSRS; Fishbach et al., 2003). This scale asks individuals (1) how successful they are in watching their weight, (2) how successful they are in losing weight, and (3) how difficult it is for them to stay in shape. These three items are answered on a 7-point scale ranging from *“Not successful at all”* (1) to *“Very successful”* (7), or *“Not difficult at all”* (1) to *“Very difficult”* (7). An average score of the three items, with the third item reverse coded, provides a general perceived dieting success score. Participants also have the option of answering with “*Not applicable”* (8) since the items do not seem applicable to individuals who do not have an intention to lose weight (Meule et al., 2012). Internal consistency of the questionnaire in the current study was questionable when excluding individuals who used answer option 8 for one or more questions (*n* = 227; Cronbach’s alpha = 0.65). Internal consistency in previous studies was comparable ranging from 0.65 to 0.72 (Meule et al., 2012). Furthermore, since leaving an item out did not improve internal consistency, the average score was used. Higher scores reflect more perceived dieting success.

#### Executive Control

Executive control was measured with the Attentional Network Task (ANT; Fan et al., 2002). A behavioral measure was used since this provides an index of actual ability instead of individuals’ own interpretation of their ability, which have been shown to be two largely unrelated constructs (e.g., Toplak et al., 2013). The ANT is a reaction time (RT) task during which participants have to determine the direction (left or right) of an arrow that is presented on the screen. This arrow is flanked by two arrows on each side, that are either pointing in the same direction (congruent) of pointing in the opposite direction (incongruent). The task consisted of 144 trials, of which 72 were congruent and 72 were incongruent. The experimental trials were preceded by 24 practice trials.

For reasons of consistency, following Macleod et al. (2010), participants with more than 30% errors on the ANT (*n* = 20) were removed from further analyses. Additionally, following Jonker et al. (2016a), before EC scores were calculated, RT’s of trials with incorrect responses (4.9%), and trials without a response (0.4%) were removed. Additionally, outlying trials (>2.5 SD from the mean: 2.6%) were removed. Subsequently, the EC score was calculated by subtracting the mean RTs on congruent trials from the mean RTs on incongruent trials. Consequently, a lower score reflects better EC (Fan et al., 2002). Internal consistency of the EC measure was acceptable (Spearman–Brown = 0.71).

### Procedure

The current study reports data that was collected as part of a combined data collection procedure of different projects. Data of the others projects have not (yet) been published. One of these projects was interested in both males and females and therefore both participated in the data collection procedure. The study was approved by the ethical committee of the psychology department of the University of Groningen (16011-S). Participants performed the study online via Qualtrics, in the language corresponding to the language of their study program (40% Dutch and 60% English). Participants signed an online informed consent form, and answered the BIS/BAS, followed by the ANT, the restraint scale, and the PSRS. Participants received study credits for their participation. To obtain study credits students are free to choose the studies they participate in. Participants who failed to correctly answer two control questions “This is a control question; click on the most left answering option,” and “This is a control question: click on the most right answering option,” were excluded (*n* = 5).

### Statistical Analyses

To examine whether PS is positively related to restrained eating (hypothesis 1) hierarchical regression analyses were performed with restrained eating as dependent variable, and PS as independent variable (step 1). In keeping with Jonker et al. (2016a) the association between restrained eating and RS and EC were also explored. Therefore, RR (model 1) or RDr (model 2) were entered in step 2, EC in step 3, and the interaction between RR × EC (model 1) or RDr × EC (model 2) was entered in step 4.

To examine whether restrained eaters’ perceived dieting success was positively related to RS (hypothesis 2a), and/or negatively related to RS in individuals low in EC (hypothesis 2b) two hierarchical regression analyses were performed with perceived dieting success as dependent variable (model 3 and 4). In keeping with model 1 and model 2, BIS was entered as independent variable (step 1). In step 2, RR (model 3) or RDr (model 4) were entered, in step 3 EC was entered, and in step 4 the interaction between RR × EC (model 3) or RDr × EC (model 4) was entered. Since we were interested in examining the association between RS and PS and perceived dieting success of restrained eaters, individuals who used answer option 8 in the PSRS scale were excluded from these analyses (model 3 and 4) since they likely do not have a dieting intention (Meule et al., 2012).

Since we tested two models (RR and RDr) with every dependent variable (restrained eating and dieting success), we used a corrected alpha of 0.025 (α of 0.05/2). To obtain 95% power to find a medium effect size within the current study at least 147 participants should be included. However, since for one of the projects more participants had to be included the current study will report on the data of all participants that meet our criteria. All independent variables were centered before they were entered in the model. The assumptions of the multiple regression analyses – no multicollinearity, a linear model, homoscedasticity, and normal distribution of the residuals – were checked, and any deviations from these assumptions were reported.

## Results

### Descriptive Statistics

After excluding participants with outliers on the ANT (*n* = 20) and who answered the control questions wrong (*n* = 5), the final sample consisted of 290 female undergraduate students. Descriptive statistics of the sample are shown in Table 1. As a result we have 95% power to find an effect of 0.07 (small to medium) or larger with an alpha of 0.025 in the regression analyses.

**TABLE 1:** Sample characteristics.

	**Total sample (*N* = 290)**			**Dieters (*N* = 227)**		
	**Mean**	**SD**	**Range**	**Mean**	**SD**	**Range**
Age	19.48	1.75	17–29	19.49	1.75	17–29
BMI	22.30	2.83	18.5–38.0	22.71	2.91	18.6–38.1
Restrained eating	13.72	5.35	1.0–29.0	14.99	5.01	1.0–29.0
PSRS	4.30	1.58	1.0–7.7	3.74	1.18	1.0–7.0
RR	3.38	0.37	2.2–4.0	3.38	0.38	2.2–4.0
RDr	2.67	0.56	1.0–4.0	2.67	0.56	1.0–4.0
PS	3.16	0.49	1.6–4.0	3.19	0.49	1.6–4.0
Executive Control	113.04	32.05	45.5–232.6	113.87	33.18	45.5–232.6

*PSRS, perceived dieting success; RR, reward responsiveness; RDr, reward drive; PS, punishment sensitivity.*

Bivariate correlational analyses (see Table 2) showed that restrained eating was related a higher BMI. Furthermore, restrained eating was weakly positively related to RR, RDr, and PS. In the group of individuals who report to be dieters restrained eating was weakly related to less perceived dieting success and a higher BMI. Perceived dieting success in dieters was weakly related to higher RDr, RR, and PS. EC was not related to restrained eating, perceived dieting success, or PS in either sample. Higher RR was weakly related to higher EC.

**TABLE 2:** Bivariate correlations between all continuous variables.

	**Total sample (*N* = 290)**						**Dieters (*N* = 227)**					
	**RE**	**PSRS**^*b*^	**BMI**	**RR**	**RDr**	**PS**	**RE**	**PSRS**	**BMI**	**RR**	**RDr**	**PS**
PSRS	–	–	–	–	–	–	−0.14*	–	–	–	–	–
BMI	0.33**	–	–	–	–	–	0.22*	−0.34**	–	–	–	–
RR	0.12*	–	0.05	–	–	–	0.14*	0.15*	0.06	–	–	–
RDr	0.15**	–	0.10	0.50**	–	–	0.17*	0.25**	0.11	0.50**	–	–
PS	0.16*	–	0.07	0.08	−0.17**	–	0.17**	−0.19**	0.07	0.11	−0.15*	–
EC^*a*^	0.03	–	0.06	−0.13*	−0.11	0.01	0.00	0.01	0.03	−0.14*	−0.12	−0.03

*RE, restrained eating; PSRS, perceived dieting success; RR, reward responsiveness; RDr, reward drive; PS, punishment sensitivity; EC, executive control.*

*^*a*^A lower EC-score reflects better executive control.*

^*b*^Scores of the PSRS including individuals who answered an item with not applicable are not interpretable.

*p < 0.05, **p < 0.01.

#### Are PS and RS Related to Restrained Eating?

Punishment sensitivity was positively related to restrained eating (see Table 3). Model 2 showed that RDr was positively related to restrained eating. No association was found between RR and restrained eating. The interaction between RS and EC did not explain additional variance in restrained eating.

**TABLE 3:** Hierarchical regression analyses.

**Dependent variable**	**Model**	**Step**	**Variable**	***B***	**SEB**	**β**	***t***	***p***	**F-change**	**Adj-R^2^** (%)
Restrained eating (*N* = 290)		1	PS	1.72	0.63	0.16	2.72	0.007*	7.41	2
	
	1	2	RR	1.60	0.84	0.11	1.90	0.058	3.62	3
		3	EC	0.01	0.01	0.05	0.88	0.380	0.77	3
		4	RR × EC	−0.04	0.02	−0.10	−1.68	0.095	2.80	4
	
	2	2	RDr	1.82	0.55	0.19	3.28	0.001*	10.13	5
		3	EC	0.01	0.01	0.06	1.00	0.319	1.00	5
		4	RDr × EC	0.01	0.02	0.03	0.55	0.587	0.30	5
		1	PS	−0.46	0.16	−0.19	−2.92	0.004*	8.55	3
	
Perceived dieting success (*N* = 227)	3	2	RR	0.42	0.20	0.17	2.59	0.010*	6.72	6
		3	EC	0.00	0.00	0.03	0.39	0.701	0.15	5
		4	RR × EC	0.01	0.01	0.10	1.46	0.147	2.12	6
	
	4	2	RDr	0.47	0.14	0.22	3.44	0.001*	11.83	8
		3	EC	0.00	0.00	0.03	0.44	0.658	0.20	7
		4	RDr × EC	0.00	0.00	0.03	0.52	0.605	0.27	7

*p < 0.025.

*RR, reward responsiveness; RDr, reward drive; PS, punishment sensitivity; EC, executive control.*

#### Are PS and RS Related to Subjective Dieting Success of Dieters?

Model 3 and 4 show that RR and RDr were positively related to perceived dieting success. Model 3 and 4 show that the interaction between RS and EC did not explain additional variance in perceived dieting success of dieters. PS was negatively related to perceived dieting success.

## Discussion

The current study was set out to improve our understanding of the characteristics of individuals who are motivated to restrict their food intake to lose weight yet who nevertheless fail to do so. We examined whether PS was related to restrained eating, and RS to restrained eaters’ perceived dieting success. The main findings can be summarized as follows: (1) PS was positively related to restrained eating, and (2) RS was positively related to perceived dieting success of dieters. Additionally, there was no evidence that EC moderated the association between RS and perceived dieting success, and PS was negatively related to perceived dieting success.

A positive association was found between restrained eating and PS. This finding is in line with previously found positive relationships between PS and restrained eating (Ahern et al., 2010; Matton et al., 2013; Jonker et al., 2016a; Matton et al., 2017b). An important next step is to examine the direction of this relationship using a longitudinal approach to examine whether high PS is a precursor of dieting behavior. Interestingly, results further showed that PS was negatively related to perceived dieting success. This seems to indicate that PS might be related to *more* intention to diet, yet *less* success at these attempts. However, since high PS has been suggested to be related to a tendency to perceive actions as incorrect or flawed (Wierenga et al., 2014), it might also be that individuals with high PS only perceive their attempts as less successful. Future studies should include a more objective measure of dieting success to further examine this association. A longitudinal study that examines the relationship between PS and weight measured over a period of time would provide insight into the association between PS and more objective dieting success. Additionally, it could be helpful to explore whether the found associations are moderated by the dieting strategy that individuals use. That is, it could be that the association between PS and dieting intention is specifically apparent in individuals who would use a rigid dieting strategy (i.e., all or nothing dieting) which is related to less dieting success than a flexible dieting approach (Westenhoefer et al., 2013).

A positive association was found between RS and perceived dieting success of dieters. EC was not found to moderate this association between RS and dieting success. The current findings seem in line with the theory in which RS is suggested to have a *facilitating* role in food restriction (Bergh and Södersten, 1996), and not in line with the theory in which RS is suggested to have an *impeding* role in food restriction (Davis and Woodside, 2002). Nevertheless, it seems too early to draw firm conclusions about the role of RS in successful food restriction. For example, causality cannot be established from the current study, and the suggestion that RS is positively related to dieting success since it increases the rewarding value of the effect of food restriction such as weight loss and improved body shape could not be examined since only a general measure of RS was included. Therefore, it might be important for future studies to examine whether increasing the reward value of the consequences of food restriction increases individual’s success in their dieting attempts. This would enable to test both the causality of the found association, as well as the assumption that heightened RS is implicated in dieting success by increasing the rewarding value of the specific effects of food restriction.

There are some limitations that should be taken into account when interpreting the results of this study. Firstly, the ANT was not performed in a controlled environment but online. Yet, participants with many errors or who provided wrong answers on the control questions were excluded, the average EC scores were very similar to those of our previous lab-based study (Jonker et al., 2016a), and internal consistency of the task was acceptable. Related, in line with the previous study in this field we used the ANT as measure of EC. However, the ANT assesses specifically executive attention. Before we conclude that EC is not a moderator in the relationship between RS and dieting success, future studies might want to examine the moderation effect with a broader measure (e.g., the stop-signal task cf., Dassen et al., 2018). Secondly, the current study included mainly healthy weight women (86%), and it might be that the role of RS and PS is different for overweight and obese dieters. Thirdly, a self-report measure was used to assess dieting success in general. This scale was found to be negatively related to BMI in both the current study and previous studies (Meule et al., 2011; Van Koningsbruggen et al., 2011), attesting to its validity. Nevertheless, future studies should examine whether the current findings also relate to specific dieting attempts. Fourthly, even though women with underweight were excluded from this study, it is possible that women with an eating disorder participated, potentially influencing the perceived dieting success findings. That is, since individuals with an eating disorder have been found to report higher PS and they might be more critical about their dieting success, this might have resulted in an overestimation of the association.

## Conclusion

To conclude, the current study showed a positive association between PS and restrained eating. It therefore adds to the evidence that PS is related to individuals’ motivation to restrict their food intake. Furthermore, findings showed that RS was positively related to dieting success of restrained eaters. This is consistent with the theory that suggests that heightened RS *facilitates* food restriction (Bergh and Södersten, 1996). As a next step it would be interesting to examine whether it is beneficial to individuals who want to lose weight to increase the rewarding value of the consequences of successful food restriction.

## Data Availability Statement

The raw data supporting the conclusions of this article will be made available by the authors, without undue reservation.

## Ethics Statement

This study was reviewed and approved by the Ethical Committee of the Psychology Department of the University of Groningen (16011-S). The participants provided their written informed consent to participate in this study.

## Author Contributions

All authors listed have made a substantial, direct and intellectual contribution to the work, and approved it for publication.

## Conflict of Interest

The authors declare that the research was conducted in the absence of any commercial or financial relationships that could be construed as a potential conflict of interest.

## References

[B1] Ahern A. L. Field M. Yokum S. Bohon C. Stice E. (2010). Relation of dietary restraint scores to cognitive biases and reward sensitivity. *Appetite* 55 61–68. 10.1016/j.appet.2010.04.001 20399819

[B2] Baumeister R. F. Vohs K. D. Tice D. M. (2007). The strenght model of self-control. *Curr. Dir. Psychol. Sci.* 16 351–355. 10.11250/chemotherapy1953.30.Supplement1_466

[B3] Bergh C. Södersten P. (1996). Anorexia nervosa, self-starvation and the reward of stress. *Nat. Med.* 2 21–22. 10.1038/nm0196-21 8564826

[B4] Carver C. S. White T. L. (1994). Behavioral inhibition, behavioral activation, and affective responses to impending reward and punishment: the BIS/BAS scales. *J. Pers. Soc. Psychol.* 67 319–333. 10.1037//0022-3514.67.2.319

[B5] Dalley S. E. (2016). Does the salience of possible selves mediate the impact of approach and avoidance temperaments on women’s weight-loss dieting? *Pers. Individ. Dif.* 88 267–271. 10.1016/j.paid.2015.09.010

[B6] Dassen F. C. M. Houben K. Allom V. Jansen A. (2018). Self-regulation and obesity: the role of executive function and delay discounting in the prediction of weight loss. *J. Behav. Med.* 41 806–818. 10.1007/s10865-018-9940-9 29802535 PMC6209053

[B7] Davis C. Fox J. (2008). Sensitivity to reward and body mass index (BMI): evidence for a non-linear relationship. *Appetite* 50 43–49. 10.1016/j.appet.2007.05.007 17614159

[B8] Davis C. Woodside D. B. (2002). Sensitivity to the rewarding effects of food and exercise in the eating disorders. *Compre. Psychiatry* 43 189–194. 10.1053/comp.2002.32356 11994836

[B9] De Decker A. Sioen I. Verbeken S. Braet C. Michels N. de Henauw S. (2016). Associations of reward sensitivity with food consumption, activity pattern, and BMI in children. *Appetite* 100 189–196. 10.1016/j.appet.2016.02.028 26898320

[B10] De Ridder D. Adriaanse M. Evers C. Verhoeven A. (2014). Who diets? Most people and especially when they worry about food. *Appetite* 80 103–108. 10.1016/j.appet.2014.05.011 24845781

[B11] Fan J. McCandliss B. D. Sommer T. Raz A. Posner M. I. (2002). Testing the efficiency and independence of attentional networks. *J. Cogn. Neurosci.* 14 340–347. 10.1162/089892902317361886 11970796

[B12] Field A. E. Aneja P. Austin S. B. Shrier L. A. de Moor C. Gordon-Larsen P. (2007). Race and gender differences in the association of dieting and gains in BMI among young adults. *Obesity* 15 456–464. 10.1038/oby.2007.560 17299119

[B13] Fishbach A. Friedman R. S. Kruglanski A. W. (2003). Leading us not unto temptation: momentary allurements elicit overriding goal activation. *J. Pers. Soc. Psychol.* 84 296–309. 10.1037/0022-3514.84.2.296 12585805

[B14] Gray J. A. (1970). The psychophysiological basis of introversion-extraversion. *Behav. Res. Ther.* 8 249–266. 10.1016/0005-7967(70)90069-0 5470377

[B15] Gray J. A. McNaughton N. (2000). “An enquiry into the functions of the sopto-hippocampal system,” in *The Neuropsychology of Anxiety*, 2nd Edn, eds Mackintosh N. J. Shallice T. Treisman A. McGaugh J. L. Schacter D. Weiskrantz L. (Oxford: Oxford University Press), 1–442. 10.1093/acprof:oso/9780198522713.001.0001

[B16] Harrison A. O’Brien N. Lopez C. Treasure J. (2010). Sensitivity to reward and punishment in eating disorders. *Psychiatry Res.* 177 1–11. 10.1016/j.psychres.2009.06.010 20381877

[B17] Herman C. P. Polivy J. (1975). Anxiety, restraint and eating behaviour. *J. Abnorm. Psychol.* 84 666–672. 10.1037//0021-843X.84.6.666 1194527

[B18] Hofmann W. Baumeister R. F. Förster G. Vohs K. D. (2012). Everyday temptations: an experience sampling study of desire, conflict, and self-control. *J. Pers. Soc. Psychol.* 102 1318–1335. 10.1037/a0026545 22149456

[B19] Jappe L. M. Frank G. K. W. Shott M. E. Rollin M. D. H. Pryor T. Hagman J. O. (2011). Heightened sensitivity to reward and punishment in anorexia nervosa. *Int. J. Eat. Disord.* 44 317–324. 10.1002/eat.20815 21472750 PMC3072848

[B20] Johnson F. Pratt M. Wardle J. (2012). Dietary restraint and self-regulation in eating behavior. *Int. J. Obes.* 36 665–674. 10.1038/ijo.2011.156 21829162

[B21] Jonker N. C. Bennik E. C. de Jong P. J. (2016a). Reinforcement sensitivity and restrained eating: the moderating role of executive control. *Eat. Weight Disord.* 23 321–329. 10.1007/s40519-016-0343-z 27888468 PMC5959999

[B22] Jonker N. C. Glashouwer K. A. Hoekzema A. Ostafin B. D. de Jong P. J. (2020). Heightened self-reported punishment sensitivity, but no differential attention to cues signaling punishment or reward in anorexia nervosa. *PLoS One* 15:e0229742. 10.1371/journal.pone.0229742 32126134 PMC7053765

[B23] Jonker N. C. Glashouwer K. A. Ostafin B. D. van Hemel-Ruiter M. E. Smink F. R. E. Hoek H. W. (2016b). Attentional bias for reward and punishment in overweight and obesity: the TRAILS study. *PLoS One* 11:e0157573. 10.1371/journal.pone.0157573 27391017 PMC4938215

[B24] Jonker N. C. Ostafin B. D. Glashouwer K. A. van Hemel-Ruiter M. E. de Jong P. J. (2014). Reward and punishment sensitivity and alcohol use: the moderating role of executive control. *Addict. Behav.* 39 945–948. 10.1016/j.addbeh.2013.12.011 24389069

[B25] Knäuper B. Cheema S. Rabiau M. Borten O. (2005). Self-set dieting rules: adherence and prediction of weight loss success. *Appetite* 44 283–288. 10.1016/j.appet.2005.01.008 15896878

[B26] Macleod J. W. Lawrence M. A. McConnell M. M. Eskes G. A. Klein R. M. Shore D. I. (2010). Appraising the ANT: psychometric and theoretical considerations of the attention network test. *Neuropsychology* 24 637–651. 10.1037/a0019803 20804252

[B27] Matton A. de Jong P. Goossens L. Jonker N. Van Malderen E. Vervaet M. (2017a). Sensitivity for cues predicting reward and punishment in young women with eating disorders. *Eur. Eat. Disord. Rev.* 25 501–511. 10.1002/erv.2541 28944522

[B28] Matton A. Goossens L. Braet C. Vervaet M. (2013). Punishment and reward sensitivity: are naturally occurring clusters in these traits related to eating and weight problems in adolescents? *Eur. Eat. Disord. Rev.* 21 184–194. 10.1002/erv.2226 23426856

[B29] Matton A. Goossens L. Vervaet M. Braet C. (2017b). Effortful control as a moderator in the association between punishment and reward sensitivity and eating styles in adolescent boys and girls. *Appetite* 111 177–186. 10.1016/j.appet.2017.01.002 28065593

[B30] Meule A. Papies E. K. Kübler A. (2012). Differentiating between successful and unsuccessful dieters. Validity and reliability of the perceived self-regulatory success in dieting scale. *Appetite* 58 822–826. 10.1016/j.appet.2012.01.028 22329988

[B31] Meule A. Westenhöfer J. Kübler A. (2011). Food cravings mediate the relationship between rigid, but not flexible control of eating behavior and dieting success. *Appetite* 57 582–584. 10.1016/j.appet.2011.07.013 21824503

[B32] Neimeijer R. A. M. de Jong P. J. Roefs A. (2013). Temporal attention for visual food stimuli in restrained eaters. *Appetite* 64 5–11. 10.1016/j.appet.2012.12.013 23280401

[B33] Oginsky M. F. Ferrario C. R. (2019). Eating “junk food” has opposite effects on intrinsic excitability of nucleus accumbens core neurons in obesity-susceptible versus -resistant rats. *J. Neurophysiol.* 122 1264–1273. 10.1152/jn.00361.2019 31365322 PMC6766731

[B34] Sala M. Egbert A. H. Lavender J. M. Goldschmidt A. B. (2018). Affect, reward, and punishment in anorexia nervosa: a narrative overview. *Eat. Weight Disord.* 23 731–737. 10.1007/s40519-018-0588-9 30288725 PMC7479630

[B35] Stapleton P. Whitehead M. (2014). Dysfunctional eating in an australian community sample: the role of emotion regulation, impulsivity, and reward and punishment sensitivity. *Aust. Psychol.* 49 358–368. 10.1111/ap.12070

[B36] Strack F. Deutsch R. (2004). Reflective and impulsive determinants of social behavior. *Pers. Soc. Psychol. Rev.* 8 220–247. 10.1207/s15327957pspr0803 15454347

[B37] Toplak M. E. West R. F. Stanovich K. E. (2013). Practitioner review: do performance-based measures and ratings of executive function assess the same construct? *J. Child Psychol. Psychiatry* 54 131–143. 10.1111/jcpp.12001 23057693

[B38] Van Koningsbruggen G. M. Stroebe W. Papies E. K. Aarts H. (2011). Implementation intentions as goal primes: boosting self-control in tempting environments. *Goal Confl. Model Eat. Behav.* 557 129–140. 10.4324/9781315141817

[B39] van Strien T. Frijters J. E. R. Bergers G. P. A. Defares P. B. (1986). The Dutch eating behavior questionnaire (DEBQ) for assessment of restrained, emotional, and external eating behavior. *Int. J. Eat. Disord.* 5 295–315. 10.1002/1098-108X(198602)5:2<295::AID-EAT2260050209<3.0.CO;2-T

[B40] Verbeken S. Braet C. Lammertyn J. Goossens L. Moens E. (2012). How is reward sensitivity related to bodyweight in children? *Appetite* 58 478–483. 10.1016/j.appet.2011.11.018 22138702

[B41] Walther M. Hilbert A. (2016). Temperament dispositions, problematic eating behaviours and overweight in adolescents. *Eur. Eat. Disord. Rev.* 24 19–25. 10.1002/erv.2381 26104832

[B42] Wardle J. Haase A. M. Steptoe A. (2006). Body image and weight control in young adults: international comparisons in university students from 22 countries. *Int. J. Obes.* 30 644–651. 10.1038/sj.ijo.0803050 16151414

[B43] Westenhoefer J. Engel D. Holst C. Lorenz J. Peacock M. Stubbs J. (2013). Cognitive and weight-related correlates of flexible and rigid restrained eating behaviour. *Eat. Behav.* 14 69–72. 10.1016/j.eatbeh.2012.10.015 23265405

[B44] Wierenga C. E. Ely A. Bischoff-Grethe A. Bailer U. F. Simmons A. N. Kaye W. H. (2014). Are extremes of consumption in eating disorders related to an altered balance between reward and inhibition? *Front. Behav. Neurosci.* 8:410. 10.3389/fnbeh.2014.00410 25538579 PMC4260511

[B45] World Health Organisation (2018). *Obesity and Overweight.* Geneva: World Health Organisation.

